# Predicting futile recanalization, malignant cerebral edema, and cerebral herniation using intelligible ensemble machine learning following mechanical thrombectomy for acute ischemic stroke

**DOI:** 10.3389/fneur.2022.982783

**Published:** 2022-09-28

**Authors:** Weixiong Zeng, Wei Li, Kaibin Huang, Zhenzhou Lin, Hui Dai, Zilong He, Renyi Liu, Zhaodong Zeng, Genggeng Qin, Weiguo Chen, Yongming Wu

**Affiliations:** ^1^Department of Radiology, Nanfang Hospital, Southern Medical University, Guangzhou, China; ^2^Department of Neurology, The Second Hospital of Jilin University, Changchun, China; ^3^Department of Neurology, Nanfang Hospital, Southern Medical University, Guangzhou, China; ^4^Hospital Office, Ganzhou People's Hospital, Ganzhou, China; ^5^Hospital Office, Ganzhou Hospital-Nanfang Hospital, Southern Medical University, Ganzhou, China

**Keywords:** acute ischemic stroke, machine learning, futile recanalization, malignant cerebral edema, cerebral herniation

## Abstract

**Purpose:**

To establish an ensemble machine learning (ML) model for predicting the risk of futile recanalization, malignant cerebral edema (MCE), and cerebral herniation (CH) in patients with acute ischemic stroke (AIS) who underwent mechanical thrombectomy (MT) and recanalization.

**Methods:**

This prospective study included 110 patients with premorbid mRS ≤ 2 who met the inclusion criteria. Futile recanalization was defined as a 90-day modified Rankin Scale score >2. Clinical and imaging data were used to construct five ML models that were fused into a logistic regression algorithm using the stacking method (LR-Stacking). We added the Shapley Additive Explanation method to display crucial factors and explain the decision process of models for each patient. Prediction performances were compared using area under the receiver operating characteristic curve (AUC), F1-score, and decision curve analysis (DCA).

**Results:**

A total of 61 patients (55.5%) experienced futile recanalization, and 34 (30.9%) and 22 (20.0%) patients developed MCE and CH, respectively. In test set, the AUCs for the LR-Stacking model were 0.949, 0.885, and 0.904 for the three outcomes mentioned above. The F1-scores were 0.882, 0.895, and 0.909, respectively. The DCA showed that the LR-Stacking model provided more net benefits for predicting MCE and CH. The most important factors were the hypodensity volume and proportion in the corresponding vascular supply area.

**Conclusion:**

Using the ensemble ML model to analyze the clinical and imaging data of AIS patients with successful recanalization at admission and within 24 h after MT allowed for accurately predicting the risks of futile recanalization, MCE, and CH.

## Introduction

Stroke is a leading cause of mortality and disability worldwide. The global deaths caused by ischemic stroke increased by 60.68% over 30 years, from 2,049,670 in 1990 to 3,293,400 in 2019 (1). Acute ischemic stroke (AIS) is characterized by a sudden reduction or cessation of blood flow in a brain artery that results in ischemia and hypoxia of the brain tissue in the corresponding blood supply area. According to current international guidelines and related research, endovascular mechanical thrombectomy (MT) combined with recombinant tissue-type plasminogen activator (rt-PA) thrombolysis is the standard treatment in patients with AIS due to occlusion of the proximal anterior intracranial region, while MT is one of the most important forms of endovascular treatment (EVT) for large vessel occlusion (2–4).

However, despite recent improvements in MT procedure, futile recanalization, defined as a 90-day modified Rankin Scale (mRS-90) score >2 after adequate vessel recanalization, remains a serious clinical problem (5). The incidence of futile recanalization after MT is approximately 49–67% (5). The primary risk factors for patients with AIS include large infarct volume, poor collateral circulation, and high National Institutes of Health Stroke Scale (NIHSS) score (6–8). While the mRS and NIHSS scores are among the methods used to evaluate AIS functional outcomes, few studies have focused on the functional outcomes and potentially lethal complications in patients with AIS who have undergone an MT and for whom recanalization was achieved. Although computed tomography-angiography and magnetic resonance imaging (MRI) can be used to accurately evaluate the entire ischemic lesion (core and penumbra), non-contrast computed Tomography (NCCT) is common for patients with AIS after MT, due to its widespread availability, low cost, and rapid scanning speed (9).

Malignant cerebral edema (MCE) and cerebral herniation (CH) are relatively common and serious complications that lead to rapid deterioration of patient's condition, coma, poor prognosis, or even death. Therefore, being able to rapidly recognize which patients are at high risk for futile recanalization and potentially lethal complications after an MT can help clinicians make individualized treatment decisions.

The machine learning (ML) method can accurately process complex nonlinear relationships among a large number of variables, which is difficult to accomplish with traditional statistical models (10, 11). This technology has been applied to predict the outcomes of patients with AIS; however, a drawback of complex ML algorithms is its interpretability has limitations, which are commonly referred to as black-box models for clinicians. Previous researchers have attempted to solve this problem using simple ML algorithms, but more complex and improved models, such as the support vector machine (SVM), deep neural network, and ensemble ML algorithms, which may perform better in stroke-related tasks have not been fully utilized (12, 13). In addition, few studies have focused on the ability of applied complex ML methods to predict the occurrence of malignant complications in patients who undergo MT and recanalization.

Therefore, in this study, ensemble ML models were constructed to predict futile recanalization, MCE, and CH in patients with AIS treated with MT and in whom successful recanalization was achieved. The model we constructed can accurately identify and display the high-risk factors of each patient.

## Methods

### Study population

**Figure 1:**
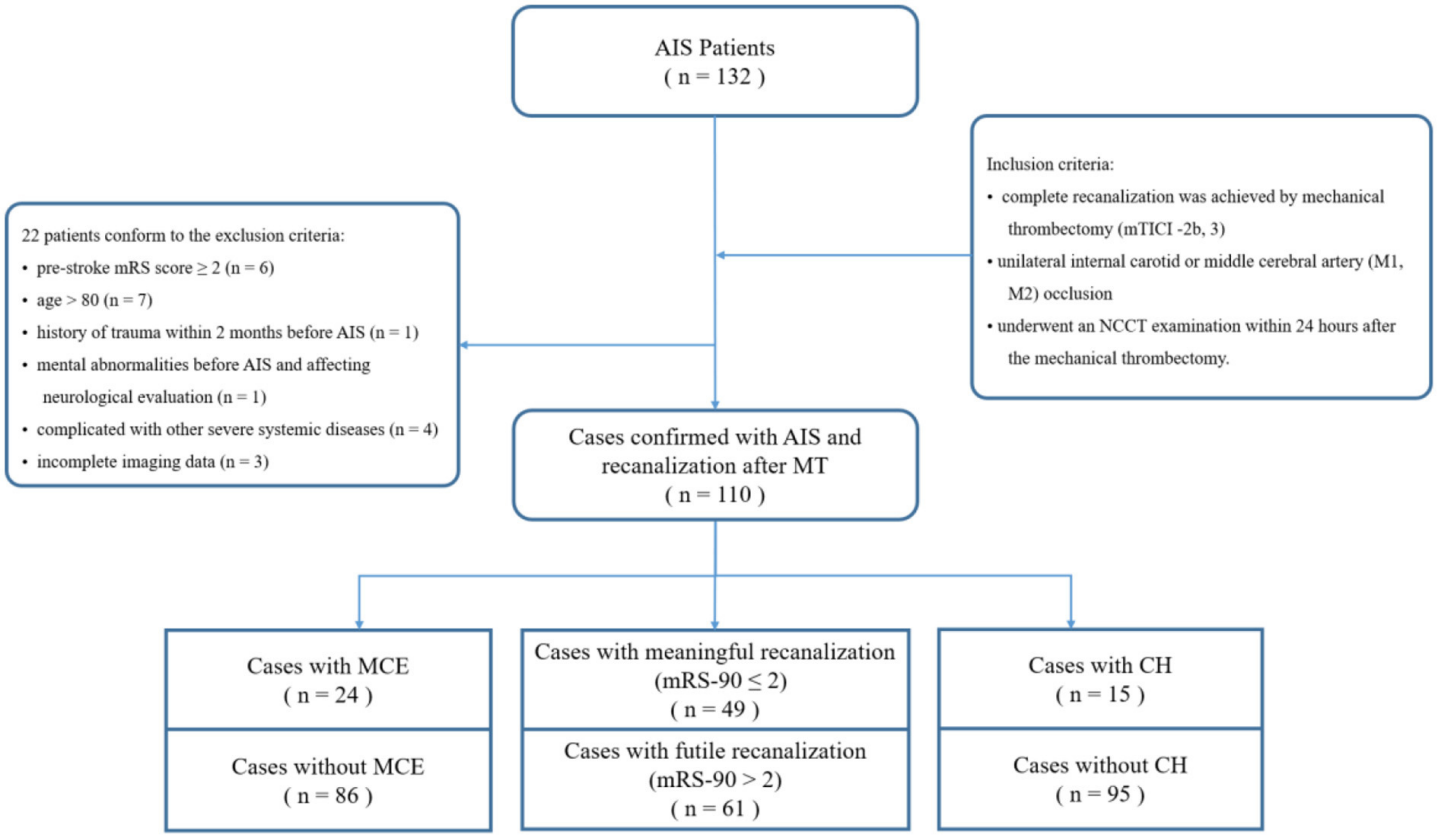
The inclusion and exclusion criteria.

We recruited 110 patients with confirmed AIS and large vessel anterior circulation occlusion who underwent MT and in whom successful recanalization was achieved, modified Thrombolysis in Cerebral Infarction (mTICI) score 2b-3, in the Department of Neurology at Nanfang Hospital between June 2016 and November 2019. All the included patients had a unilateral internal carotid or middle cerebral artery (M1, M2) occlusion that was confirmed using digital subtraction angiography. A femoral artery puncture was performed within 6 h of stroke onset unless the ischemic and infarction areas were mismatched found by imaging evaluation (CTP and MRA) and MT was deemed necessary; the puncture could be performed within 6–24 h. The patients underwent an NCCT examination within 24 h after the MT. Figure 1 shows the inclusion and exclusion criteria. The decision to perform MT and administer rt-PA was made individually for each patient through a consensus of therapeutic neurologists and neurointerventionalists and by following national and international guidelines (3). The exclusion criteria were as follows: (1) age >80 years; (2) premorbid mRS >2; (3) history or evidence of cerebral hemorrhage, subarachnoid hemorrhage, venous malformations, or brain aneurysms or tumors; (4) high risk of bleeding, such as platelet count < 100 × 109/L, active bleeding, trauma, or surgery within 2 months before the onset of stroke; (5) mental abnormalities before stroke that affected neurological function assessments; (6) comorbid hematological conditions, malignant tumors, severe heart, lung, liver, renal failure, or life expectancy of < 1 year.

### Image acquisition and feature extraction

Using the NCCT scan that was acquired for each patient with AIS, we calculated the volume (mm^3^) and maximum area (mm^2^) of the hypo- and hyperdense lesions on the picture archiving and communication system workstation using manual segmentation and automatic measurement tools. All the images were independently studied by two experienced neurologists who were blinded to the clinical characteristics. Differences of opinion were resolved through discussion. The proportion of hypodense lesions in the responsible vascular supply area was categorized into one of the following four levels: 0: no hypodense lesions; 1: proportion < 1/3; 2: proportion between 1/3 and 2/3; 3: proportion >2/3. The proportion of hyperdense lesions in the responsible vascular supply area was categorized into one of the following four levels: 0: no hyperdense lesions; 1: scattered punctate hyperdensity lesions were observed; 2: fused hyperdensity, but the area was < 1/3 of the corresponding vascular supply area, with or without a space-occupying effect; 3: fused hyperdensity and area >1/3 of the corresponding vascular supply area, with or without a space-occupying effect (14, 15). We also observed hyperdensity in the subarachnoid space and calculated the Alberta Stroke Program Early CT Score (ASPECTS) based on the NCCT images that were acquired at admission and within 24 h after the MT (16).

### Clinical assessments and outcomes

Baseline demographic and clinical characteristics (sex, age, smoking, NIHSS score, Glasgow Coma Scale (GCS) score, blood pressure, and blood sugar on admission), history of cardiovascular diseases (hypertension, hyperlipidemia, coronary heart disease, atrial fibrillation, and diabetes), time from stroke onset to femoral artery puncture, and thrombolytic therapy were each considered in the present study. The feature set also included interventional surgical-related characteristics (time interval from stroke onset to vascular recanalization, duration of surgery, thrombolysis or not, and times of embolectomy), and blood testing results before and after MT (D-dimer, fibrinogen, leukocytes, neutrophils, and lymphocytes).

The mRS-90 is used to indicate a patient's functional outcome; therefore, meaningful recanalization was defined in this study as mRS-90 of 0–2, and futile recanalization was defined as mRS-90 of 3–6. We used the same feature set to predict the risk of MCE and CH. MCE was defined as meeting the following two criteria: (1) an increase in the NIHSS score ≥4 or an increase of the consciousness evaluation part of the NIHSS score ≥1; (2) the range of the hypodense lesions was >50% of the supply area of the middle cerebral artery, and it was accompanied by signs of local brain edema, such as lateral ventricle compression, disappearance of the sulcus, midline displacement of the septum pellucidum, or a pineal layer >5 mm with basal cistern occlusion (17). CH was defined as meeting the following two criteria: (1) one or more of the following clinical symptoms occur the presence of vomiting: decreased consciousness, or mydriasis with the disappearance of the light reflex; (2) CT- or MRI-confirmed brain tissue displacement (18).

### Model development

A dataset was constructed, which included baseline demographic and clinical characteristics, clinical information before and after interventional surgery, and brain NCCT features after MT. To preprocess the data, the missing dataset values were filled by averages calculated based on the complete dataset, and the dataset was randomly divided into a training set and a test set at a ratio of 7:3. We normalized the quantitative data to a 0–1 range to accelerate and improve model training. When the level of each indicator varies greatly, the role of the indicator with high value in the comprehensive analysis will be highlighted, and the role of the indicator with a low-value level will be relatively weakened. Data standardization can effectively prevent gradient explosion and overfitting (19, 20). The multiclassification data were processed using one-hot encoding. To solve the problem of the unbalanced sample size of the patients with MCE and CH, we used the upsampling method, synthetic minority oversampling technique (SMOTE), to balance the training dataset (21). The SMOTE algorithm is implemented by imblearn package in Python 3.7.4.

In the present study, five common ML algorithms, including SVM, random forest classifier (RFC), extreme gradient boosting (XGBoost), *k*-nearest neighbor (KNN), and gradient-boosting machine (GBM), were developed and validated using the scikit-learn and XGBoost packages in Python 3.7.4 to predict futile recanalization, MCE, and CH in the patients with AIS. Ten-fold cross-validation was used for model derivation and internal validation. The grid search algorithm was used during the training process for each model to optimize model's hyperparameters on the training set as the standard of the area under the receiver operating characteristic curve (AUC).

**Figure 2:**
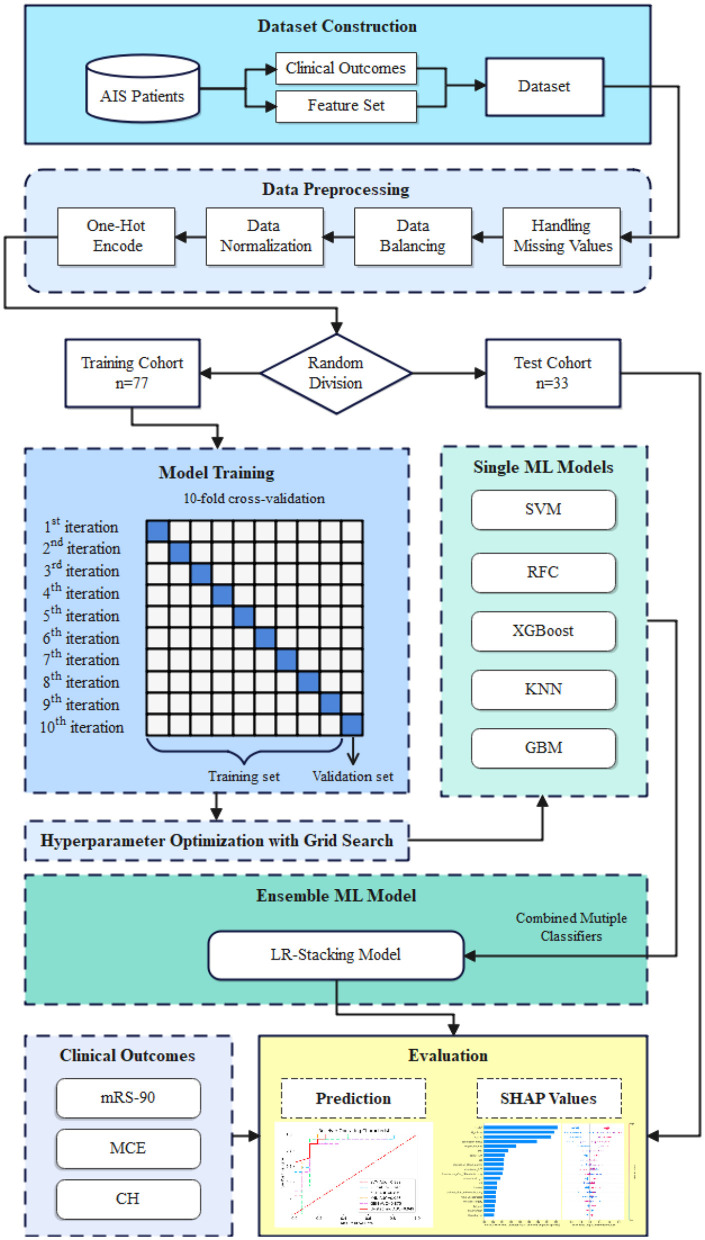
The model development pipeline. First, data were randomly divided into training and test sets without duplication. Next, using the training set, the five basic ML algorithms were internally trained, and their predictive ability was validated by applying a 10-fold cross-validation and hyperparameter optimization using the grid search method. Subsequently, the basic ML models were integrated into the LR-Stacking model, and the optimal model was evaluated in test set.

We used the five basic ML models as base learners and developed a stacking ensemble model using the logistic regression (LR-Stacking) algorithm as the meta-learner. The model development pipeline is shown in Figure 2 and the detailed process for constructing the LR-Stacking model is shown in Supplementary Figure 1.

### Model evaluation

The AUC, sensitivity, specificity, accuracy, and F1-score of the five basic ML models and LR-Stacking model were calculated in the test set, and used to assess the performance of the models. The superiority of the ensemble ML algorithm over the conventional statistical method was evaluated by comparing the performance of the ensemble ML models and LR model. The AUCs of the models were compared using the Delong test in MedCalc 19.0.7 (MedCalc Software Ltd., Ostend, Belgium).

The Shapley Additive Explanations (SHAP) local explanatory technique explained the optimal model by calculating each feature's contribution to the predictive results individually and globally (22, 23). According to this model interpretation method, the feature importance of each prediction task can be observed, and the basis of the prediction results obtained by the model for each patient.

### Statistical analysis

Univariate analyses were performed using the Mann–Whitney *U* test for continuous variables and the chi-squared test for categorical variables. All the tests were two-sided, and statistical significance was set at *P* < 0.05. Statistical analyses were performed using SPSS version 25.0 (IBM Corp., Armonk, NY, USA) and R Studio 4.0.3 (R Foundation for Statistical Computing, Vienna, Austria).

## Results

### Study population

A total of 110 patients with AIS (average age, 58.16 ± 12.57 years; 78 males and 32 females) were included in this study. Among them, 61 (55.5%) patients experienced futile recanalization, 34 (30.9%) developed MCE, and 22 (20.0%) developed CH. The dataset was randomly divided into a training set (*n* = 77, 70%) and a test set (*n* = 33, 30%). In the training set, there were 44 (57.1%) patients with futile recanalization, 24 (31.2%) with MCE, and 15 (19.5%) with CH. In the test set, there were 17 (51.5%) patients with futile recanalization, 10 (30.3%) with MCE, and 7 (21.2%) with CH. For MCE, SMOTE algorithm generated 29 cases in the training set, including 19 MCE and 10 non-MCE. For CH, SMOTE algorithm generated 47 cases with CH in the training set. The demographic data including the generated data are shown in Supplementary Tables 1–3.

Table 1 displays several significant differences in characteristics across the two groups of meaningful recanalization and futile recanalization. The patients with futile recanalization had lower GCS scores at admission, higher D-dimer levels after undergoing embolectomy, lower ASPECTS within 24 h after embolectomy, and greater prevalence of hyperdensity in subarachnoid than meaningful recanalization. The complete characteristic distribution differences among the three groups are shown in Supplementary Tables 4–6. The patients with MCE were older, and they had lower GCS scores and ASPECTS at admission, higher D-dimer, WBC, and neutrophil levels, and a higher frequency of the large artery atherosclerosis (LAA) TOAST classification than non-MCE patients. The patients with CH had a shorter interval from onset to puncture, lower ASPECTS at admission, and higher D-dimer, WBC, and neutrophil levels than non-CH. Furthermore, the patients with AIS and either futile recanalization, MCE, or CH had broad hyper- and hypodense lesions, and they generally accounted for a large proportion of the responsible vascular supply area.

**Table 1:** Summary of the important characteristics comparing AIS patients with futile recanalization vs. meaningful recanalization.

	**mRS-90 ≤ 2 (Mean-sd/IQR/N)**	**mRS-90 > 2 (Mean-sd/IQR/N)**	**All (Mean-sd/IQR/N)**	***p*-value**
Patients	49 (44.5%)	61 (55.5%)	110	
Age	56.04 ± 13.08	59.87 ± 11.98	58.16 ± 12.57	0.144
NIHSS at admission	11.73 (2–23)	15.62 (3–28)	13.89 (2–28)	0.059
GCS at admission	12.67 (6–15)	10.94 (3–15)	11.71 (3–15)	0.021*
DBP	79.29 ± 16.07	80.95 ± 12.25	80.21 ± 14.04	0.284
Blood glucose at admission	6.44 (5.62–8.55)	7.21 (6.50–8.82)	7.11 (6.11–8.69)	0.168
TOAST-LAA	19	23	42	0.354
Hyperdensity proportion				< 0.001*
0	32	23	55	
1	12	5	17	
2	5	17	22	
3	0	16	16	
Hyperdensity volume	0 (0–1.43)	2.90 (0–13.19)	0.20 (0–4.55)	< 0.001*
ASPECTS after embolectomy	9.43 (7–10)	8.38 (4–10)	8.85 (4–10)	0.029*
Hyperdensity in subarachnoid	11	27	38	0.017*
Hyperdensity in anyposition	23	43	66	0.012*
Maximum slice area of hyperdensity	0 (0–95.48)	260.98 (0–1097.02)	15.26 (0–430.41)	< 0.001*
Hypodensity proportion				< 0.001*
1	40	14	54	
2	4	16	20	
3	5	31	36	
Hypodensity proportion > 2/3	5	31	36	< 0.001*
Hypodensity proportion > 1/3	9	47	56	< 0.001*
Hypodensity volume	15.16 (5.21–31.98)	97.81 (34.43–177.63)	39.79 (12.67–127.83)	< 0.001*
D-dimer after embolectomy	1.31 (0.78–2.57)	3.18 (1.48–6.86)	2.25 (1.02–5.52)	0.004*

mRS-90, 90-day modified Rankin Scale; NCCT, non-contrast computed tomography; ASPECTS, alberta stroke program early CT score.

^*^Significant difference between the two groups (p < 0.05).

### Model performance

**Figure 3:**
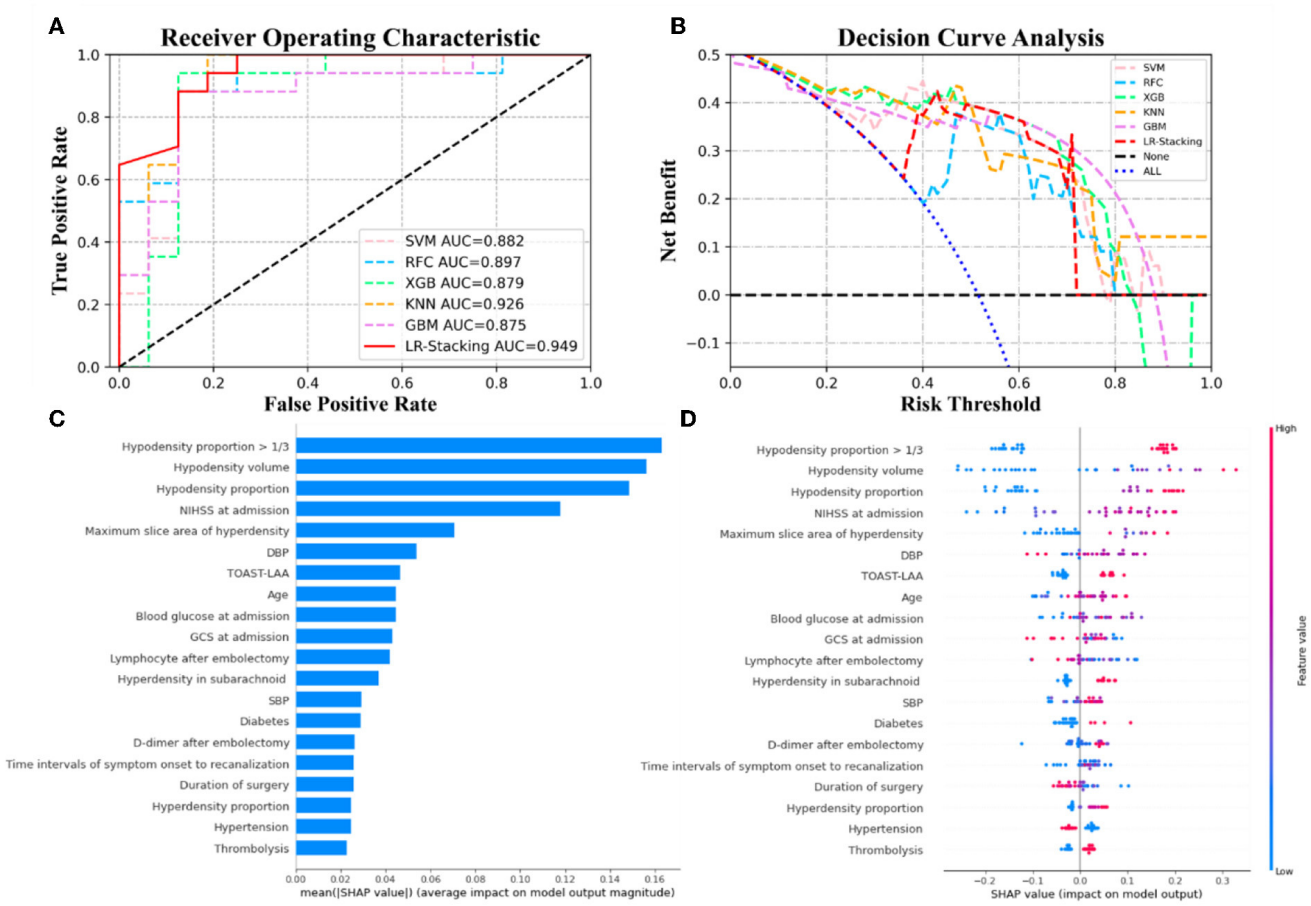
The results from the ML models and contributions of various features to predicting futile recanalization. **(A)** The ROC curve of five ML models and LR-Stacking model. **(B)** The net benefit of the various models. **(C)** The features are listed in descending order according to the contributions from the LR-Stacking model. **(D)** The effects of the features on prediction. The colors indicate the value of each feature, from high (red) to low (blue). The horizontal location shows whether the effect of the value leads to a prediction of futile recanalization. Each point is a SHAP value of a feature for a case.

**Figure 4:**
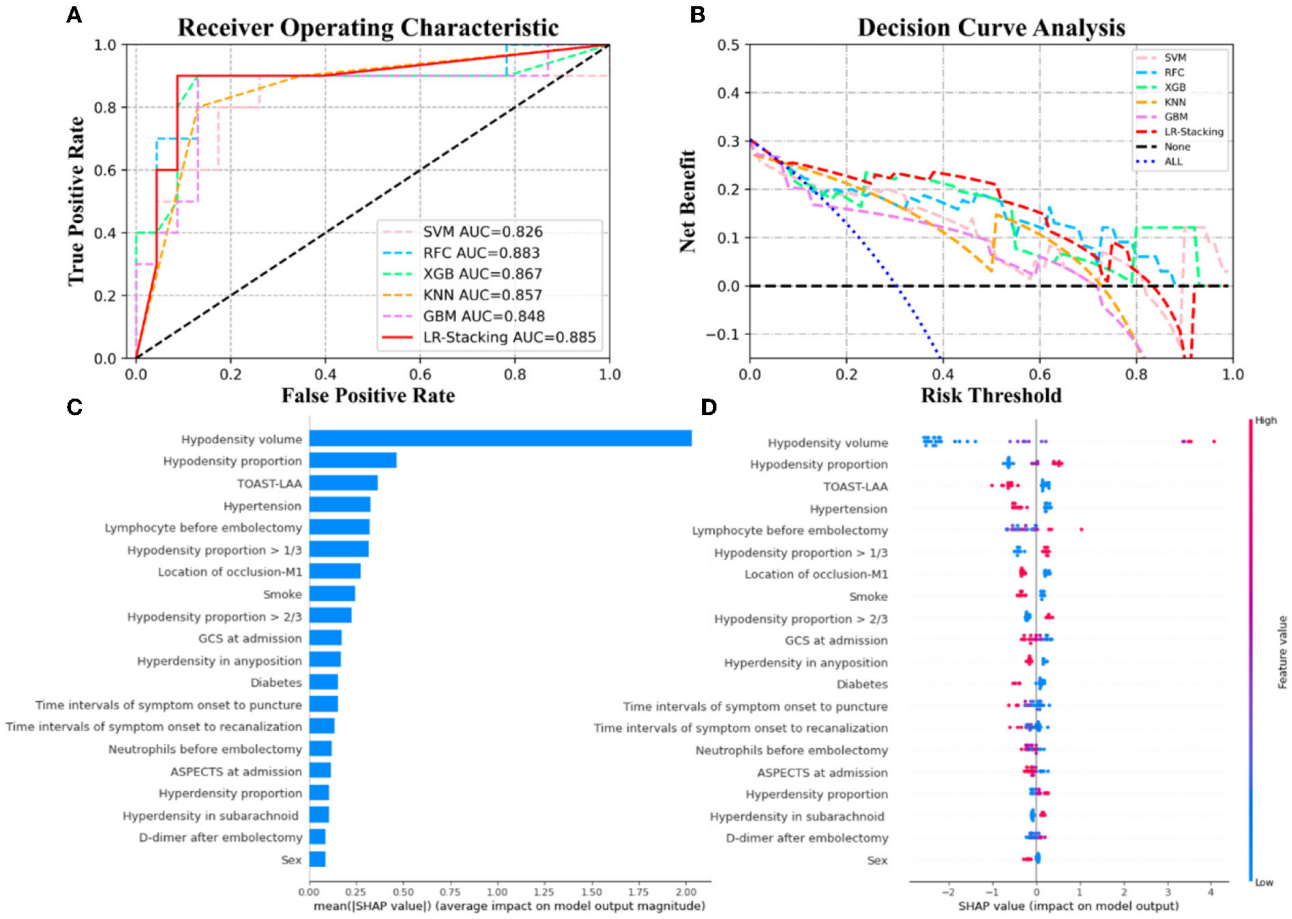
The results of the ML models and the contributions of various features to predicting MCE. **(A)** The ROC curve of five ML models and LR-Stacking model. **(B)** The net benefit of the various models. **(C)** The importance of the features for the LR-Stacking model. **(D)** The effects of the features on the predictions of the LR-Stacking model.

**Figure 5:**
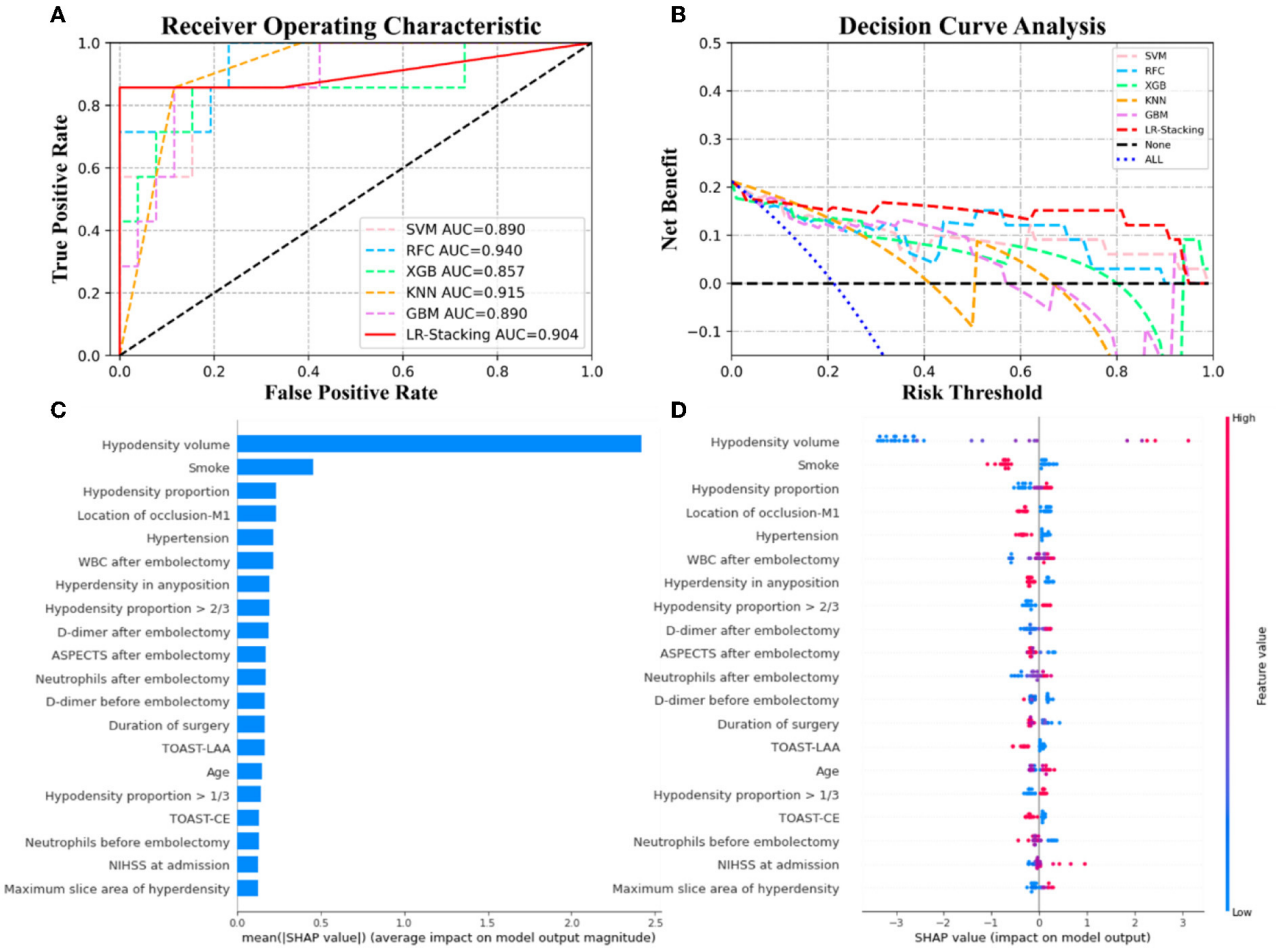
The results of the ML models and contributions of various features to predicting CH. **(A)** The ROC curve of five ML models and LR-Stacking model. **(B)** The net benefit of the various models. **(C)** The importance of the features for the LR-Stacking model. **(D)** The effects of the features on the predictions of the LR-Stacking model.

Each basic ML algorithm performed well in the binary category classification of mRS-90, MCE, and CH. The AUC, sensitivity, specificity, accuracy, and F1-score of each model using the independent test set are presented in Table 2. Figures 3–5 show the receiver operating characteristic curve (ROC), decision curve analysis (DCA), and feature importance of each basic ML and LR-Stacking model for the three classification tasks. The optimal basic ML models (KNN, RFC, and RFC) predicting futile recanalization, MCE, and CH had AUCs of 0.927, 0.883, and 0.940, respectively, sensitivities of 88.2, 90.0, and 71.4%, respectively, specificities of 87.5, 87.0, and 96.2%, respectively, accuracies of 87.9, 87.9, and 90.9%, respectively, and F1-scores of 0.879, 0.864, and 0.856, respectively.

**Table 2:** The AUC, sensitivity, specificity, accuracy, and F1-score comparisons.

	**AUC**	**Sensitivity**	**Specificity**	**Accuracy**	**F1-score**
**mRS-90**					
SVM	0.882 (0.751, 1.000)	0.882 (0.64, 0.99)	0.875 (0.617, 0.985)	0.879 (0.718, 0.966)	0.879
RFC	0.897 (0.782, 1.000)	0.882 (0.64, 0.99)	0.813 (0.544, 0.960)	0.849 (0.681, 0.949)	0.857
XGBoost	0.879 (0.734, 1.000)	0.882 (0.64, 0.99)	0.875 (0.617, 0.985)	0.879 (0.718, 0.966)	0.882
KNN	0.927 (0.828, 1.000)	0.882 (0.64, 0.99)	0.875 (0.617, 0.985)	0.879 (0.718, 0.966)	0.879
GBM	0.875 (0.747, 1.000)	0.882 (0.64, 0.99)	0.813 (0.544, 0.960)	0.849 (0.681, 0.949)	0.848
LR-stacking	0.949 (0.882, 1.000)	0.882 (0.64, 0.99)	0.875 (0.617, 0.985)	0.879 (0.718, 0.966)	0.882
**MCE**					
SVM	0.826 (0.628, 1.000)	0.700 (0.348, 0.933)	0.826 (0.612, 0.951)	0.788 (0.611, 0.910)	0.756
RFC	0.883 (0.725, 1.000)	0.900 (0.555, 0.998)	0.870 (0.664, 0.972)	0.879 (0.718, 0.966)	0.864
XGBoost	0.867 (0.690, 1.000)	0.800 (0.444, 0.975)	0.913 (0.720, 0.989)	0.879 (0.718, 0.966)	0.856
KNN	0.857 (0.714, 0.999)	0.800 (0.444, 0.975)	0.870 (0.664, 0.972)	0.849 (0.681, 0.949)	0.825
GBM	0.848 (0.671, 1.000)	0.500 (0.187, 0.813)	0.870 (0.664, 0.972)	0.758 (0.577, 0.889)	0.694
LR-stacking	0.885 (0.738, 1.000)	0.900 (0.555, 0.998)	0.913 (0.720, 0.989)	0.909 (0.757, 0.981)	0.895
**CH**					
SVM	0.890 (0.756, 1.000)	0.571 (0.184, 0.901)	0.962 (0.804, 0.999)	0.879 (0.718, 0.966)	0.796
RFC	0.940 (0.851, 1.000)	0.714 (0.290, 0.963)	0.962 (0.804, 0.999)	0.909 (0.757, 0.981)	0.856
XGBoost	0.857 (0.654, 1.000)	0.571 (0.184, 0.901)	0.923 (0.749, 0.991)	0.849 (0.681, 0.949)	0.761
KNN	0.915 (0.827, 1.000)	0.857 (0.421, 0.996)	0.885 (0.699, 0.976)	0.879 (0.718, 0.966)	0.835
GBM	0.890 (0.760, 1.000)	0.714 (0.290, 0.963)	0.885 (0.699, 0.976)	0.849 (0.681, 0.949)	0.784
LR-Stacking	0.904 (0.715, 1.000)	0.857 (0.421, 0.996)	0.962 (0.804, 0.999)	0.939 (0.798, 0.993)	0.909

AUC, area under the receiver operating characteristic curve; mRS-90, 90-day modified rankin scale; MCE, malignant cerebral edema; CH, cerebral herniation; SVM, support vector machine; RFC, random forest classifier; XGBoost, extreme gradient boosting; KNN, k-nearest neighbor; GBM, gradient-boosting machine; LR, logistics regression.

For predicting the futile recanalization, MCE, and CH, the LR-Stacking models had AUCs of 0.949, 0.885, and 0.904, respectively, sensitivities of 88.2, 90.0, and 85.7%, respectively, specificities of 87.5, 91.3, and 96.2%, respectively, accuracies of 87.9, 90.9, and 93.9%, respectively, and F1-scores of 0.882, 0.895, and 0.909, respectively. Compared with the optimal basic ML models for predicting futile recanalization and MCE, the Delong test showed that the AUC of the LR-Stacking model improved by 0.022 (*p* = 0.457) and 0.002 (*p* = 0.927), respectively. For predicting CH, the AUC of the LR-Stacking model decreased by 0.036 (*p* = 0.635) compared with that of the RFC model. Moreover, the LR-Stacking models performed better than all five basic ML models in terms of their sensitivity, specificity, accuracy, and especially F1-score.

Under the same conditions, for predicting futile recanalization, MCE, and CH, the LR models had AUCs of 0.908, 0.852, and 0.929, respectively. Comparing the performance of the ensemble ML method against the generalized statistical method for predicting futile recanalization and MCE demonstrated that the AUC of the LR-Stacking model improved by 0.041 (*p* = 0.324) and 0.032 (*p* = 0.395), respectively. For predicting CH, the AUC of the LR-Stacking model decreased by 0.025 (*p* = 0.739). Similarly, the LR models show a similar trend to the basic ML models in that their accuracy, F1-score, and other statistical are lower than those of the LR-Stacking models. The model comparison results are shown in Table 3.

**Table 3:** The AUC, sensitivity, specificity, accuracy, and F1-score comparisons of generalized LR and LR-Stacking method.

	**AUC**	**Sensitivity**	**Specificity**	**Accuracy**	**F1-score**	***p*-value**
**mRS-90**						
LR	0.908 (0.7914, 1.0000)	0.882 (0.6356, 0.9854)	0.875 (0.6165, 0.9845)	0.879 (0.7180, 0.9660)	0.879	0.324
LR-stacking	0.949 (0.882, 1.000)	0.882 (0.64, 0.99)	0.875 (0.617, 0.985)	0.879 (0.718, 0.966)	0.882	
**MCE**						
LR	0.852 (0.6551, 1.0000)	0.900 (0.5550, 0.9975)	0.870 (0.6641, 0.9722)	0.879 (0.7180, 0.9660)	0.864	0.395
LR-stacking	0.885 (0.738, 1.000)	0.900 (0.555, 0.998)	0.913 (0.720, 0.989)	0.909 (0.757, 0.981)	0.895	
**CH**						
LR	0.929 (0.8263, 1.0000)	0.714 (0.2904, 0.9633)	0.923 (0.7487, 0.9905)	0.879 (0.7180, 0.9660)	0.819	0.739
LR-stacking	0.904 (0.715, 1.000)	0.857 (0.421, 0.996)	0.962 (0.804, 0.999)	0.939 (0.798, 0.993)	0.909	

The AUCs of three groups of models were compared by Delong test.

AUC, area under the receiver operating characteristic curve; mRS-90, 90-day modified Rankin Scale; MCE, malignant cerebral edema; CH, cerebral herniation; LR, logistics regression.

DCA demonstrated that if the threshold probability in the clinical decision was >20%, the ML models provided a greater benefit than the treat-all models. For classifying MCE and CH, the overall net benefit of the LR-Stacking model was greater than that of the other ML models. For example, at the 40% risk cutoff, the net benefits from the LR-Stacking model were 23 and 16%, respectively, which are equivalent to performing clinical interventions for 23 MCE patients and 16 CH patients per 100 patients without any of the interventions being unnecessary and 21 (MCE) and 25 (CH) fewer unnecessary interventions with no increase in the number of clinically significant missed MCE and CH diagnoses.

### Feature importance analysis

For predicting the outcomes of the patients with AIS, the LR-Stacking model indicated that the most important characteristics were the hypodensity volume and proportion of the responsible vascular supply area, NIHSS score at admission, and maximum layer area of hyperdensity. For predicting MCE and CH, the LR-Stacking model primarily classified patients by their hypodensity volume. The SHAP values for all the basic ML models are shown in Supplementary Figures 2–4.

**Figure 6:**
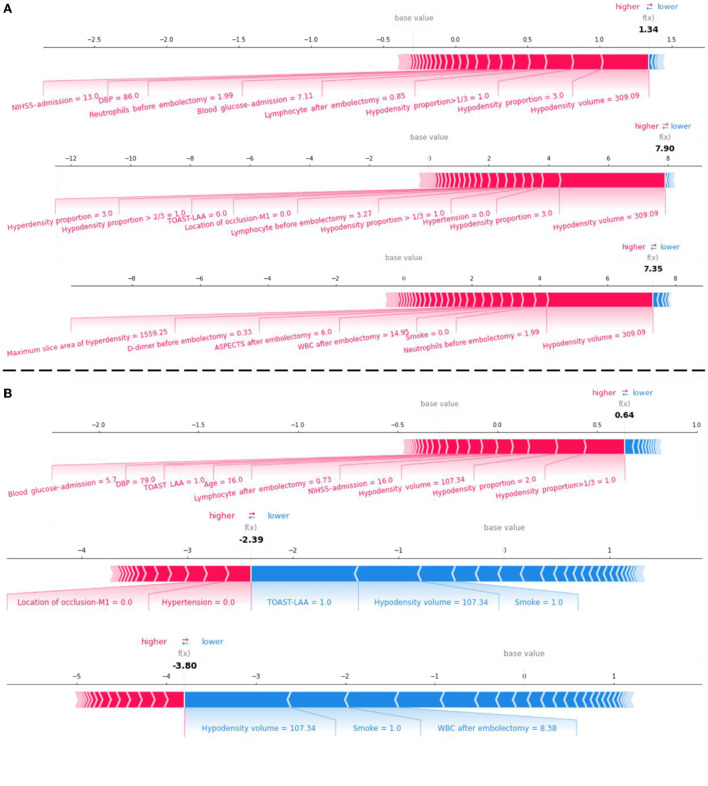
The force plot for the LR-Stacking model decision process for evaluating the risk of futile recanalization, MCE, and CH in two patients with AIS in the test set. **(A)** A patient with mRS-90 of 5, indicating futile recanalization and developed MCE and CH. **(B)** A patient with mRS-90 of 2, indicating meaningful recanalization and did not develop MCE or CH. Each feature provides a SHAP value for the base value of the model. The final prediction value, f(x), is obtained using to the weight of the features and the model processing. When f(x) > 0, the model determines that the case is positive; otherwise, it is considered negative.

We displayed the LR-Stacking models' decision-making processes for two patients from the test set. The models' prediction processes for the mRS-90, MCE, and CH are shown in Figure 6. Case #1 (Figure 6A) was a patient who had an mRS-90 of 5, indicating futile recanalization; this patient developed MCE and CH. Case #2 (Figure 6B) was a patient who had an mRS-90 of 2, indicating meaningful recanalization; this patient did not develop MCE or CH. We found that the LR-Stacking models output the classification results for case #1 primarily based on the hypodensity volume; however, the other features that supported a futile recanalization prediction were different from those for case #2. The LR-stacking model incorrectly classified case #2 as having an mRS-90 >2, primarily due to the high hypodensity proportion in the responsible vascular supply. The LR-Stacking model also determined that MCE and CH would not occur in case #2 due to the TOAST being classified as LAA and the presence of a relatively small hypodense volume.

## Discussion

The present study demonstrated that the predictive models based on clinical and NCCT- characteristics and ensemble ML algorithm allows to accurately predict the risk of futile recanalization, MCE, and CH in patients with AIS who were treated with MT and for whom successful endovascular recanalization was achieved. In terms of overall prediction performance, the ensemble ML method in predicting these three adverse events is better than that of the basic ML models and generalized statistical method. We added SHAP algorithm to show the top features and how they impact the models' output. The results of SHAP analysis showed that hypodensity volume and proportion of the responsible vascular supply area, NIHSS at admission, and maximum slice area of hyperdensity was the top-5 predictors for predicting futile recanalization. Meanwhile, hypodensity volume and proportion, TOAST-LAA were the top-3 predictors for predicting MCE, and hypodensity volume and proportion, and smoking history were the top-3predictors for predicting CH.

Most studies demonstrated that ML can be used as an auxiliary means of clinical evaluation to predict the functional outcomes after EVT of AIS patients (24–26). However, most of the research cohorts were AIS patients with anterior circulation infarction, regardless of the efficacy of EVT, and only their mRS-90 was concerned. Despite complete endovascular recanalization, a significant percentage of patients with AIS do not achieve a good clinical outcome (5). The characteristics of the present study are that it focused on patients with AIS who underwent MT and completely recanalization (mTICI score 2b-3), and three adverse outcomes including mRS-90, MCE, and CH were predicted. Moreover, the prediction models could display the specific decision-making process of each patient, which indicated that it may have the potential for clinical application. It means that the models can identify the patients with a high risk of adverse outcomes as early as possible and help doctors to be alert and take the high-risk factors suggested by the model as the target of personalized intervention.

According to the results, we can easily observe that the performance of the ensemble ML models was better than the basic ML models and the generalized LR models in the prediction of futile recanalization and MCE. Early identification of high-risk patients with MCE or CH is of great significance in treatment decisions. Prediction models require excellent sensitivity and net benefits due to the severe consequences of misclassifying MCE and CH. Although the AUC of LR-Stacking model was lower than that of RFC, we chose the LR-Stacking model as the final prediction model for evaluating the risk of CH after considering additional scores, particularly the F1-score and DCA results.

The more complex and accurate the ML model, the worse its interpretability. The primary obstacle to the application and popularization of AI prediction models in the clinical setting is the difficulty clinicians experience understanding, trusting, and using ML model prediction results and applying them to each patient. Although some ML algorithms have embedded modules of feature importance, they are still insufficient to support clinical applications. Therefore, we added SHAP algorithm to visualize the decision-making process of the ML models. According to the analysis results, we can easily observe that clinically severe AIS has a high probability of producing adverse outcomes according to the severity and extent of the initial ischemia. However, although hypodensity volume and proportion showed a strong correlation with the three adverse events, it should be emphasized that they alone were not enough to reliably complete the prediction tasks. A pooled analysis of 7 randomized multicenter trials on EVT demonstrated that only 12% of the treatment benefit according to mRS-90 could be explained by the follow-up infarct volume, which is not a valid proxy for estimating treatment effect in phase II and III trials of AIS (27). On this point, the basic ML algorithms in this study could integrate hypodensity volume and proportion and other meaningful predictors of adverse outcomes because it is good at finding and processing complex relationships between numerous input variables to make more accurate predictions (28). After that, by integrating the advantages of five basic ML algorithms, the optimal models were constructed.

Several studies have shown that a large infarct volume is associated with worse functional outcomes for patients with AIS, indicating that the infarct volume is an independent predictor of functional outcomes for these patients (29–31). Furthermore, multiple factors, including clinical and imaging features and MT-related information, may affect whether futile recanalization occurs. Analyzing these factors will help clinicians make individualized decisions about the necessity of an MT for their patients. Hypertension, LAA, older age, hyperglycemia, and lower GCS scores at admission support the model to predict poor functional prognosis. Hypertension and age over 70 may increase the risk of futile recanalization (32–34). Hyperglycemia is related to larger infarct volumes and reduced salvage of perfusion-diffusion mismatch tissue (35). On the other hand, hyperglycemia may cause a larger increase in the infarct volume leading to a worse clinical outcome despite complete recanalization (36). Functional outcomes of AIS patients after MT were similar among different TOAST subtypes, but it is still unknown whether the subtype has an impact on the patients with complete recanalization (37). Some studies also suggested that the functional outcomes of patients with LAA were worse than other TOAST criteria, which may be related to inflammation and metabolic response (38, 39). Moreover, previous studies have shown that for patients with AIS, large infarct volume, poor collateral circulation, and high NIHSS score are significant predictors of functional outcomes and indicators of the severity of the neurological injury (6–8). However, unlike in other studies, our results show that the symptom onset time and interval from puncture to recanalization did not play a particularly strong role in predicting futile recanalization (12, 40).

The infarct volume can be used to predict MCE by measuring it on early MRI scans accurately; however, MRI scans may not be readily available to patients with AIS (41). In contrast, hypodensity is easily available and measurable in CT. Although it may be a variable combination of infarction and edema, hypodensity is also closely correlated with the mRS-90, potentially lethal MCE, and CH (41–43). The SHAP values for the LR-Stacking models indicate that the NCCT-based infarct volume is an important risk factor for predicting MCE and CH. Interestingly, the model considered that the history of smoke is a protective factor against MCE and CH after MT, which was contrary to our common sense and some previous research. Smoking severely affects the cerebrovascular reserve and induces intracranial atherosclerotic changes, and it may impair cerebrovascular reactivity and lead to poor collateral circulation (44, 45). However, a meta-analysis based on 45,826 AIS patients showed a similar result that smoking was a protective factor against MCE (46). According to a relevant study, the activation of endogenous cannabinoid system may play a significant role in the neuroprotective effect of nicotine (47). It may be due to nicotine promoting the release of endocannabinoids, resulting in hypothermia, which inhibits the inflammatory response and alleviates cerebral edema (48, 49). Despite the SHAP values for other features being much lower than the infarct volumes, it cannot be assumed that other features are not essential or useful. In addition, we found that the patients with CH had shorter groin puncture time in this cohort, but there was no statistical difference in groin puncture time between the cohorts of futile recanalization and non-futile recanalization. We think it is caused by the small sample size of data because there was no special treatment performed for this group of patients before MT. This feature also did not play a significant role in our ML models. As the base learners of the LR-Stacking model, the great performances of the SVM, RFC, and KNN algorithms are facilitated by the interactions among multiple features. Overall, the NCCT-based cerebral infarct volume was the most stable and robust predictor in each basic ML model.

Our study had some limitations. First, this was a single-center study with a small sample size, and the constructed models need further external validation. Second, the low MCE and CH proportions may have affected the statistical power of the study; therefore, we applied SMOTE to the data segmentation and model training to reduce the influence of the unbalanced data. Finally, this study did not distinguish between the ischemic core and penumbra, and their impact on ML is unknown.

## Conclusion

This study demonstrates that comprehensive analysis of clinical and NCCT characteristics using ML algorithms allowed for the accurate prediction of clinical outcomes and malignant complications following MT for patients with AIS. We designed interpretable LR-stacking models constructed using five basic ML algorithms and used them as final prediction models. The hypodensity volume and proportion in the responsible vascular supply area were the most important imaging predictors, and the NIHSS score at admission was the most important clinical predictor of futile recanalization, whereas the hypodensity volume was the most important predictor of both MCE and CH. We utilized SHAP technology to show the ensemble model evaluation process for each case, which enabled us to promptly determine the individual risk factors for adverse outcomes and design corresponding clinical interventions to improve the prognosis and reduce the risk of malignant complications in the patients with AIS.

## Data availability statement

The raw data supporting the conclusions of this article will be made available by the authors, without undue reservation.

## Ethics statement

This study was approved by the Medical Ethics Committee of Nanfang Hospital (NFEC-2019-189). Informed consent was waived by the local institutional review boards due to subject anonymity and the minimal risk to the participants.

## Author contributions

WZ contributed to the experiment design, model construction, and manuscript drafting. WL contributed to the data acquisition, statistical analysis, and manuscript drafting. KH and ZL contributed to the data acquisition and image analysis. HD and RL contributed to the model construction and manuscript revisions. ZH and ZZ contributed to the statistical analysis and manuscript revisions. YW, WC, and GQ provided guidance during the entire study, including for model construction, experiment design, and critical manuscript revisions. All authors contributed to the article and approved the submitted version.

## Funding

This research was funded by the National Natural Science Foundation of China (Grant number: 82071484), the Guangdong Basic and Applied Basic Research Foundation (Grant number: 2019A1515011760), and the National Natural Science Foundation of China (Grant number: 82171929).

## Conflict of interest

The authors declare that the research was conducted in the absence of any commercial or financial relationships that could be construed as a potential conflict of interest.

## Publisher's note

All claims expressed in this article are solely those of the authors and do not necessarily represent those of their affiliated organizations, or those of the publisher, the editors and the reviewers. Any product that may be evaluated in this article, or claim that may be made by its manufacturer, is not guaranteed or endorsed by the publisher.
