# Supplementary material for: Predicting futile recanalization, malignant cerebral edema, and cerebral herniation using intelligible ensemble machine learning following mechanical thrombectomy for acute ischemic stroke
Source: Front Neurol. 2022 Sep 28;13:982783. doi: 10.3389/fneur.2022.982783 (PMC9554641; doi:10.3389/fneur.2022.982783)
Supplement: Supplementary file 1 [file Table_1.pdf]

Supplementary Material

1 Supplementary Figures

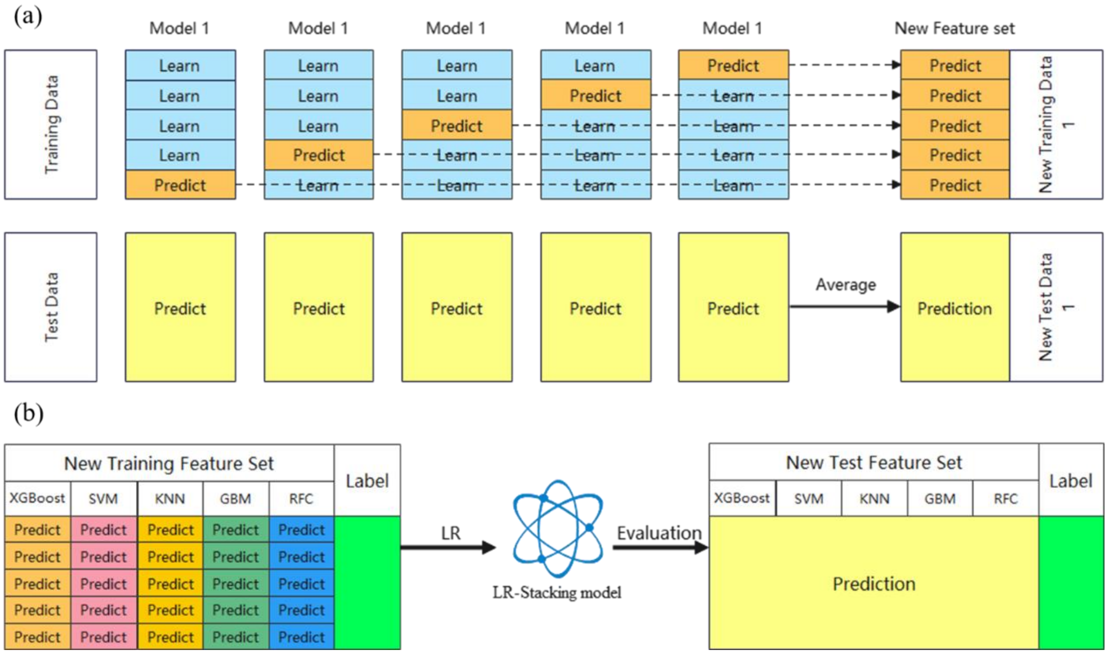

Supplementary Figure 1. Explanatory diagram for the stacking method. (a) Model 1 refers to the five ML algorithms as a base learner completing a 10-fold cross-validation. Limited by space, the figure displays the five-fold cross-validation process. Each cross-validation includes model training and internal validation based on the training data and predicting test data. The internal validation prediction results of each cross-validation round become part of the characteristics of the training set of the LR-Stacking model. Additionally, the constructed model in each cross-validation round will predict the complete test data and output the prediction results. These results are then averaged, after which they become part of the characteristics of the test set of the LR-Stacking model. (b) After repeating the above steps five times, new training and test set features that do not contain the features from within the original data can be created based on the five ML models. The new training feature sets and labels are input into the LR algorithm, and the optimal LR-Stacking model is constructed using a 10-fold cross-validation and grid search method. Finally, the LR-Stacking model is validated using the new test feature set and test set label.

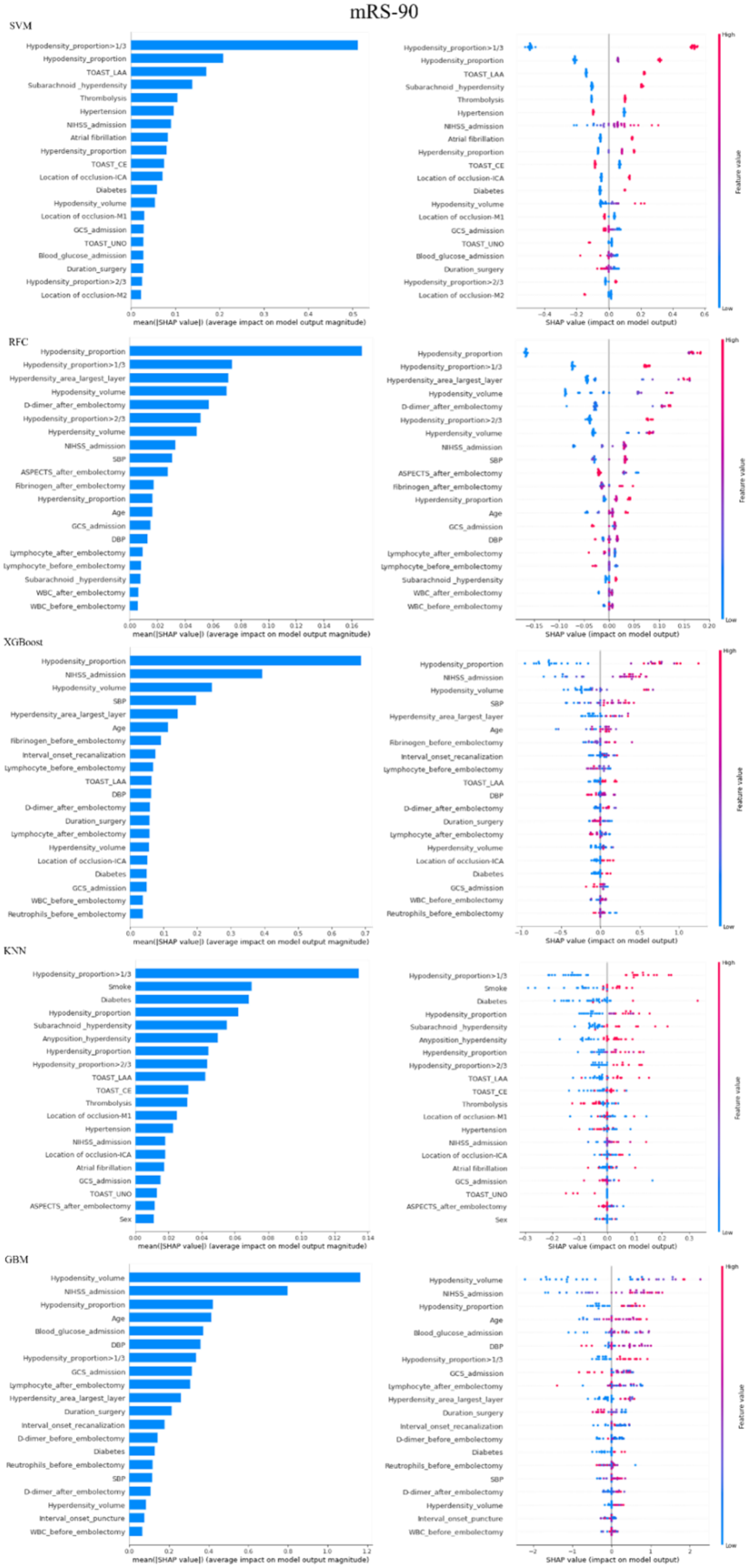

Supplementary Figure 2. The SHAP values for the five ML algorithms for predicting futile recanalization.

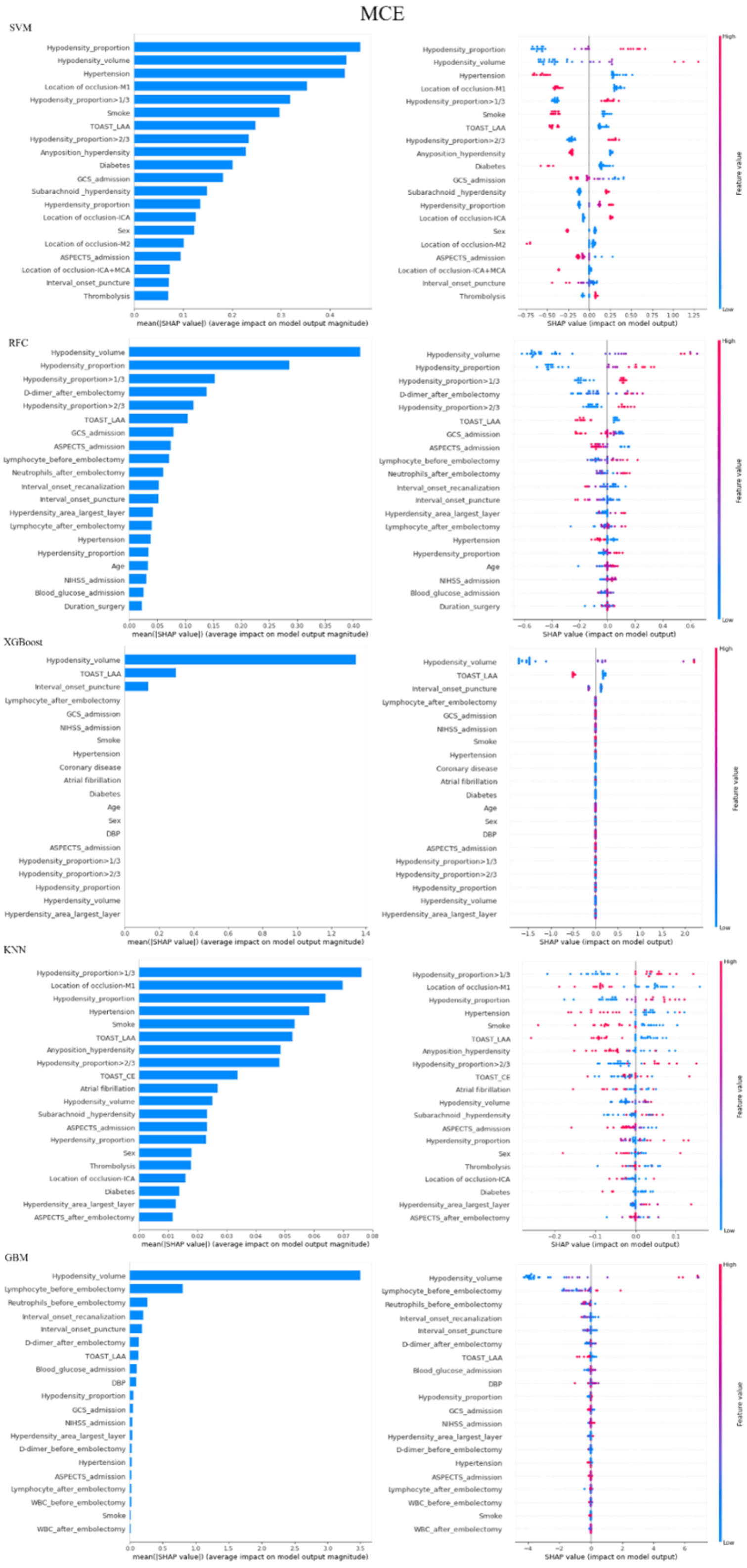

Supplementary Figure 3. The SHAP values for the five ML algorithms for predicting MCE.

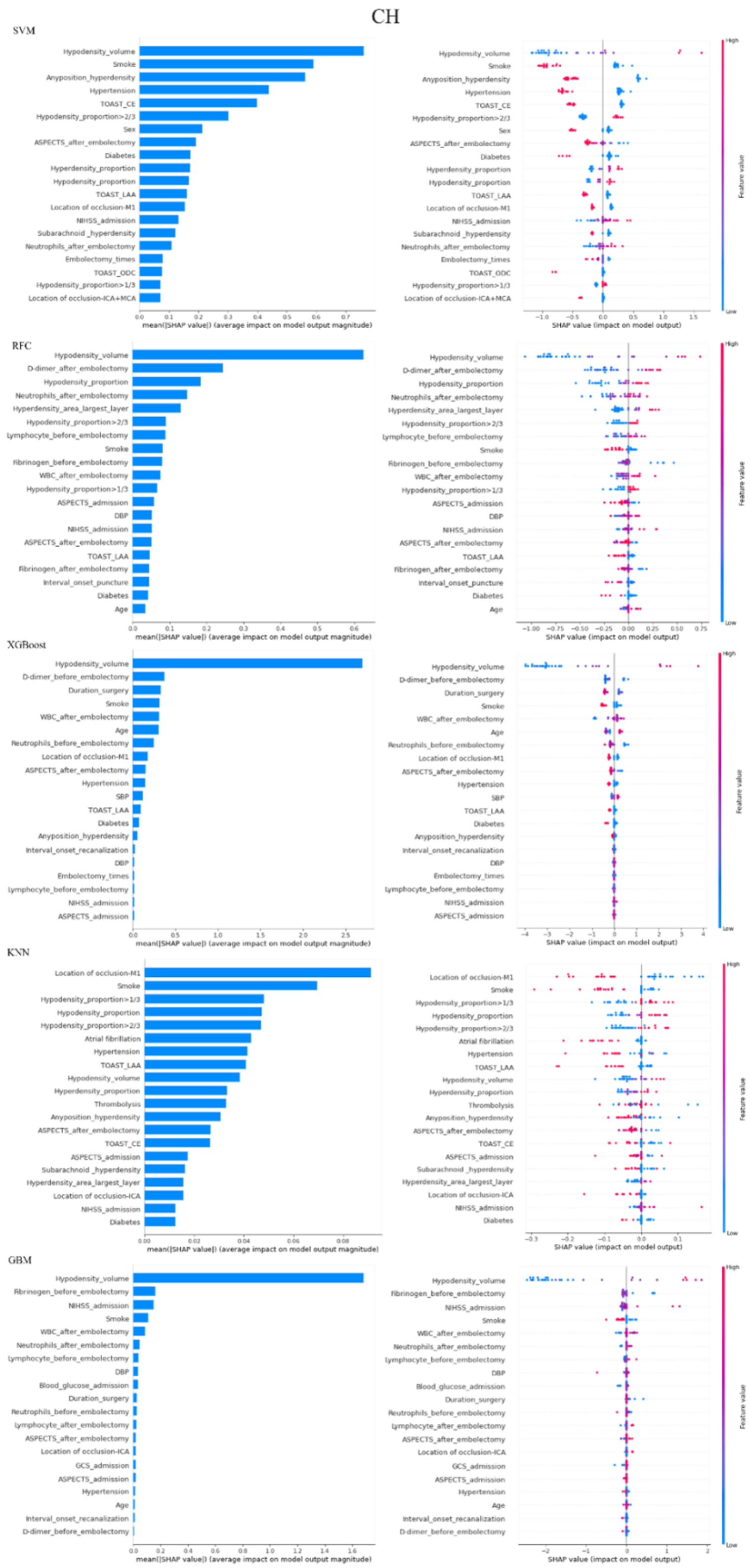

Supplementary Figure 4. The SHAP values for the five ML algorithms for predicting CH.

2 Supplementary Tables

Supplementary Table 1. The demographic data of the original training and test set.

		Training Set (Mean±sd, N%)	Test Set (Mean±sd, N%)	*p*-value
Age		58.01±12.43	58.52±13.09	0.899
Sex	Male	71.4%	69.7%	0.855
	Female	28.6%	30.3%	
Diabetes	Yes	35.1%	12.1%	0.014*
	No	64.9%	87.9%	
Atrial fibrillation	Yes	26.0%	33.3%	0.432
	No	74.0%	66.7%	
Coronary disease	Yes	13.0%	6.1%	0.286
	No	87.0%	93.9%	
Hypertension	Yes	49.4%	42.4%	0.505
	No	50.6%	57.6%	
Smoke	Yes	42.9%	51.5%	0.403
	No	57.1%	48.5%	

*Significant difference between the two groups (p <0.05).

Supplementary Table 2. The demographic data of the generated training set for MCE.

		Training Set (Mean±sd, N%)	Test Set (Mean±sd, N%)	*p*-value
Age		57.81±11.54	58.52±13.09	0.705
Sex	Male	78.3%	69.7%	0.310
	Female	21.7%	30.3%	
Diabetes	Yes	27.4%	12.1%	0.072
	No	72.6%	87.9%	
Atrial fibrillation	Yes	21.7%	33.3%	0.175
	No	78.3%	66.7%	
Coronary disease	Yes	9.4%	6.1%	0.547
	No	90.6%	93.9%	
Hypertension	Yes	39.6%	42.4%	0.774
	No	60.4%	57.6%	
Smoke	Yes	33.0%	51.5%	0.055
	No	67.0%	48.5%	

*Significant difference between the two groups (p <0.05).

Supplementary Table 3. The demographic data of the generated training set for CH.

		Training Set (Mean±sd, N%)	Test Set (Mean±sd, N%)	*p*-value
Age		60.25±11.88	58.52±13.09	0.705
Sex	Male	81.5%	69.7%	0.141
	Female	18.5%	30.3%	
Diabetes	Yes	21.8%	12.1%	0.216
	No	78.2%	87.9%	
Atrial fibrillation	Yes	16.9%	33.3%	0.038*
	No	83.1%	66.7%	
Coronary disease	Yes	9.7%	6.1%	0.517
	No	90.3%	93.9%	
Hypertension	Yes	37.1%	42.4%	0.576
	No	62.9%	57.6%	
Smoke	Yes	27.4%	51.5%	0.009*
	No	72.6%	48.5%	

*Significant difference between the two groups ($p < 0.05$).

Supplementary Table 4. Summary of the baseline epidemiological, clinical, NCCT-based, and laboratory tests characteristics comparing AIS patients with futile recanalization versus meaningful recanalization.

	mRS-90≤2 (Mean-sd/IQR/N)	mRS-90>2 (Mean-sd/IQR/N)	All (Mean-sd/IQR/N)	*p*-value	Proportion of Missing Values
Patients	49 (44.5%)	61 (55.5%)	110		
Sex				0.914	0%
Male	35	43	78		
Female	14	18	32		
Age	56.04±13.08	59.87±11.98	58.16±12.57	0.144	0%
Smoke	22	28	50	0.916	0%
NIHSS at admission	11.73 (2-23)	15.62 (3-28)	13.89 (2-28)	0.059	0%
GCS at admission	12.67 (6-15)	10.94 (3-15)	11.71 (3-15)	0.021*	0%
SBP	134.29±22.65	138.44±21.12	136.59±21.81	0.296	0%
DBP	79.29±16.07	80.95±12.25	80.21±14.04	0.284	0%
Blood glucose at admission	6.44 (5.62-	7.21 (6.50-8.82)	7.11 (6.11-8.69)	0.168	15%
Comorbidities					
Diabetes	10	21	31	0.104	0%

Atrial fibrillation	10	21	31	0.104	0%
Coronary disease	4	8	12	0.408	0%
Hypertension	21	31	52	0.406	0%
Angiographic characteristics					
Location of occlusion				0.509	0%
ICA	13	17	30		
M1	28	30	58		
M2	5	5	10		
ICA+MCA	3	9	12		
TOAST				0.354	0%
LAA	19	23	42		
CE	18	30	48		
SAO	0	0	0		
ODC	3	3	6		
UNO	9	5	14		
NCCT-based characteristics					
Hyperdensity proportion				< 0.001*	0%
0	32	23	55		
1	12	5	17		
2	5	17	22		
3	0	16	16		
Hyperdensity volume	0 (0-1.43)	2.90 (0-13.19)	0.20 (0-4.55)	< 0.001*	0%
ASPECTS after embolectomy	9.43 (7-10)	8.38 (4-10)	8.85 (4-10)	0.029*	0%
Hyperdensity in subarachnoid	11	27	38	0.017*	0%
Hyperdensity in anyposition	23	43	66	0.012*	0%
Maximum slice area of hyperdensity	0 (0-95.48)	260.98 (0-1097.02)	15.26 (0-430.41)	< 0.001*	0%
Hypodensity proportion				< 0.001*	0%
1	40	14	54		
2	4	16	20		
3	5	31	36		
Hypodensity proportion > 2/3	5	31	36	< 0.001*	0%
Hypodensity proportion > 1/3	9	47	56	< 0.001*	0%
Hypodensity volume	15.16(5.21-31.98)	97.81 (34.43-177.63)	39.79 (12.67-127.83)	< 0.001*	0%
ASPECTS at admission	8.88 (6-10)	8.36 (3-10)	8.59 (3-10)	0.436	0%
Surgical-related characteristics					
TI of symptom onset to puncture	300 (200-416)	265 (209-363)	279.5 (209.5-390)	0.362	0%
Thrombolysis	21	36	57	0.092	0%
Duration of surgery	80 (53.5-	73 (53-94)	74.50 (53-100)	0.694	0%
TI of symptom onset to recanalization	350 (282-490)	345 (300-450.5)	347 (295.75-485)	0.931	0%

Times of embolectomy	2.20 (1-6)	2.38 (1-8)	2.3 (1-8)	0.974	0%
Blood testing characteristics					
D-dimer before embolectomy	0.44 (0.28-	0.55 (0.33-1.42)	0.50 (0.33-1.30)	0.255	31%
Fibrinogen before embolectomy	2.51 (2.27-	3.17 (2.29-3.84)	2.68 (2.29-3.61)	0.118	24%
D-dimer after embolectomy	1.31 (0.78-	3.18 (1.48-6.86)	2.25 (1.02-5.52)	0.004*	40%
Fibrinogen after embolectomy	2.75±0.91	2.99±1.32	2.89±1.17	0.387	35%
WBC before embolectomy	9.19±2.81	9.43±3.05	9.31±2.92	0.469	24%
Neutrophils before embolectomy	6.80±2.82	6.82±3.00	6.81±2.90	0.897	24%
Lymphocyte before embolectomy	1.69 (1.27-	1.76 (1.23-2.19)	1.70 (1.27-2.18)	0.802	24%
WBC after embolectomy	11.33±3.67	12.28±3.72	11.88±3.71	0.114	21%
Neutrophils after embolectomy	9.24±3.23	10.49±12.76	9.98±3.47	0.056	21%
Lymphocyte after embolectomy	1.36±0.68	1.12±0.54	1.22±0.61	0.107	21%

NIHSS, National Institutes of Health Stroke Scale; GCS, Glasgow Coma Scale; ASPECTS, Alberta Stroke Program Early CT Score; SBP, systolic pressure; DBP, diastolic pressure; ICA, internal carotid artery; MCA, middle cerebral artery; TOAST, trial of ORG 10172 in acute stroke treatment; LAA, large artery atherosclerosis; CE, cardioembolism; SAO, small-artery occlusion; ODC, stroke of other determined causes; UNO, stroke of undetermined etiology; TI, Time intervals.

*Significant difference between the two groups (p <0.05).

Supplementary Table 5. Summary of the baseline epidemiological, clinical, NCCT-based, and laboratory tests characteristics comparing AIS patients with MCE versus without MCE.

	Without MCE (Mean-sd/IQR/N)	With MCE (Mean-sd/IQR/N)	*p*-value
Patients	76 (69.1%)	34 (30.9%)	
Sex			0.614
Male	55	23	
Female	21	11	
Age	56.72±12.23	61.38±12.90	0.044*
Smoke	37	13	0.309
NIHSS at admission	13.26 (2-28)	15.29 (3-26)	0.331
GCS at admission	12.16 (5-15)	10.71 (3-15)	0.007*
SBP	136.74±23.12	136.26±18.89	0.951
DBP	79.47±15.04	81.85±11.51	0.181
Blood glucose at admission	7.09 (5.68-8.54)	7.30 (6.49-9.00)	0.162
Comorbidities			
Diabetes	22	9	0.790
Atrial fibrillation	18	13	0.117

Coronary disease	9	3	0.639
Hypertension	37	15	0.658
Angiographic characteristics			
Location of occlusion			0.792
ICA	20	10	
M1	42	16	
M2	7	3	
ICA+MCA	7	5	
TOAST			0.025*
LAA	35	7	
CE	27	21	
SAO	0	0	
ODC	3	3	
UNO	11	3	
NCCT-based characteristics			
Hyperdensity proportion			<0.001*
0	42	13	
1	16	1	
2	16	6	
3	2	14	
Hyperdensity volume	0 (0-2.97)	3.85 (0-16.24)	0.002*
ASPECTS after embolectomy	9.17 (6-10)	8.12 (4-10)	0.016*
Hyperdensity in subarachnoid	20	18	0.007*
Hyperdensity in anyposition	43	23	0.274
Maximum slice area of hyperdensity	0 (0-251.93)	535.38 (0-1566.39)	0.001*
Hypodensity proportion			<0.001*
1	52	2	
2	15	5	
3	9	27	
Hypodensity proportion > 2/3	9	27	<0.001*
Hypodensity proportion > 1/3	24	32	<0.001*
Hypodensity volume	21.45 (6.65-50.72)	163.33 (105.13-262.72)	<0.001*
ASPECTS at admission	8.91 (6-10)	7.88 (3-10)	0.012*
Surgical-related characteristics			
TI of symptom onset to puncture	295.00 (220.75-421.50)	250.00 (207.50-325.25)	0.058
Thrombolysis	36	21	0.163
Duration of surgery	74.50 (53.50-112.75)	76.50 (52.75-93.25)	0.741
TI of symptom onset to recanalization	295.00 (220.75-421.50)	207.50 (250.00-325.25)	0.139
Times of embolectomy	2.33 (1-8)	2.24 (1-6)	0.906
Blood testing characteristics			

D-dimer before embolectomy	0.50 (0.30-1.19)	0.50 (0.33-1.42)	0.409
Fibrinogen before embolectomy	2.67 (2.37-3.54)	2.83 (2.08-4.02)	0.969
D-dimer after embolectomy	1.48 (0.81-2.94)	5.54 (2.53-8.44)	<0.001*
Fibrinogen after embolectomy	2.96±0.94	2.76±1.50	0.264
WBC before embolectomy	9.32±2.90	9.29±3.01	0.635
Neutrophils before embolectomy	6.98±2.99	6.45±2.71	0.719
Lymphocyte before embolectomy	1.62 (1.24-1.96)	2.11 (1.33-2.29)	0.131
WBC after embolectomy	11.14±3.57	13.37±3.59	0.003*
Neutrophils after embolectomy	9.22±3.23	11.49±3.49	0.003*
Lymphocyte after embolectomy	1.24±0.62	1.18±0.61	0.608

NIHSS, National Institutes of Health Stroke Scale; GCS, Glasgow Coma Scale; ASPECTS, Alberta Stroke Program Early CT Score; SBP, systolic pressure; DBP, diastolic pressure; ICA, internal carotid artery; MCA, middle cerebral artery; TOAST, trial of ORG 10172 in acute stroke treatment; LAA, large artery atherosclerosis; CE, cardioembolism; SAO, small-artery occlusion; ODC, stroke of other determined causes; UNO, stroke of undetermined etiology; TI, Time intervals.

*Significant difference between the two groups ($p < 0.05$).

Supplementary Table 6. Summary of the baseline epidemiological, clinical, NCCT-based, and laboratory tests characteristics comparing AIS patients with CH versus without CH.

	Without CH (Mean-sd/IQR/N)	With CH (Mean-sd/IQR/N)	*p*-value
Patients	88 (80%)	22 (20%)	
Sex			0.401
Male	64	14	
Female	24	8	
Age	57.37±12.09	61.32±14.19	0.087
Smoke	44	6	0.056
NIHSS at admission	13.40 (2-28)	15.86 (10-26)	0.716
GCS at admission	11.89 (3-15)	11.00 (7-15)	0.071
SBP	136.68±22.434	136.23±19.60	0.908
DBP	79.56±14.55	82.82±11.67	0.158
Blood glucose at admission	7.15 (6.03-8.58)	7.11 (6.19-8.82)	0.529
Comorbidities			
Diabetes	26	5	0.525
Atrial fibrillation	22	9	0.138
Coronary disease	9	3	0.646
Hypertension	43	9	0.504

Angiographic characteristics			
Location of occlusion			0.657
ICA	24	6	
M1	48	10	
M2	8	2	
ICA+MCA	8	4	
TOAST			0.072
LAA	38	4	
CE	33	15	
SAO	0	0	
ODC	5	1	
UNO	12	2	
NCCT-based characteristics			
Hyperdensity proportion			<0.001*
0	46	9	
1	17	0	
2	20	2	
3	5	11	
Hyperdensity volume	0 (0-3.10)	6.00 (0-16.24)	0.031*
ASPECTS after embolectomy	9.09 (6-10)	7.86 (4-10)	0.001*
Hyperdensity in subarachnoid	28	10	0.229
Hyperdensity in anyposition	53	13	0.922
Maximum slice area of hyperdensity	0 (0-271.54)	723.80 (0-1597.34)	0.009*
Hypodensity proportion			<0.001*
1	53	1	
2	19	1	
3	16	20	
Hypodensity proportion > 2/3	16	20	<0.001*
Hypodensity proportion > 1/3	35	21	<0.001*
Hypodensity volume	9.82 (0-25.02)	200.22 (149.87-287.299)	<0.001*
ASPECTS at admission	8.78 (4-10)	7.82 (3-10)	0.014*
Surgical-related characteristics			
TI of symptom onset to puncture	295.00 (220.75-420.50)	249.50 (199.75-282.50)	0.042*
Thrombolysis	45	12	0.775
Duration of surgery	80 (55-109)	70 (50-82.75)	0.220
TI of symptom onset to recanalization	370.00 (300.50-498.25)	315.00 (284.75-402.00)	0.052
Times of embolectomy	2.28 (1-8)	2.36 (1-6)	0.870
Blood testing characteristics			
D-dimer before embolectomy	0.51 (0.32-1.25)	0.40 (0.33-1.42)	0.906
Fibrinogen before embolectomy	2.67 (2.34-3.61)	2.83 (1.85-3.60)	0.547

D-dimer after embolectomy	1.73 (0.88-3.39)	6.94 (3.38-10.88)	<0.001*
Fibrinogen after embolectomy	2.92±1.06	2.80±1.50	0.492
WBC before embolectomy	9.26±3.00	9.52±2.85	0.555
Neutrophils before embolectomy	6.91±3.01	6.44±2.44	0.956
Lymphocyte before embolectomy	1.68 (1.27-2.18)	2.11 (1.28-2.18)	0.268
WBC after embolectomy	11.23±3.67	14.40±2.69	<0.001*
Neutrophils after embolectomy	9.29±3.34	12.61±2.67	<0.001*
Lymphocyte after embolectomy	1.23±0.62	1.16±0.58	0.718

NIHSS, National Institutes of Health Stroke Scale; GCS, Glasgow Coma Scale; ASPECTS, Alberta Stroke Program Early CT Score; SBP, systolic pressure; DBP, diastolic pressure; ICA, internal carotid artery; MCA, middle cerebral artery; TOAST, trial of ORG 10172 in acute stroke treatment; LAA, large artery atherosclerosis; CE, cardioembolism; SAO, small-artery occlusion; ODC, stroke of other determined causes; UNO, stroke of undetermined etiology; TI, Time intervals.

*Significant difference between the two groups ($p < 0.05$).
